# Analysis of microplastics in the environment: Identification and quantification of trace levels of common types of plastic polymers using pyrolysis-GC/MS

**DOI:** 10.1016/j.mex.2023.102143

**Published:** 2023-03-21

**Authors:** Lúcia H.M.L.M. Santos, Sara Insa, Marta Arxé, Gianluigi Buttiglieri, Sara Rodríguez-Mozaz, Damià Barceló

**Affiliations:** a Catalan Institute for Water Research (ICRA-CERCA), C/ Emili Grahit 101, Girona 17003, Spain; b University of Girona, Girona, Spain; c Department of Environmental Chemistry, IDAEA-CSIC, C/ Jordi Girona 18-26, Barcelona 08034, Spain

**Keywords:** Pyrolysis, GC/MS, Plastic pollution, Environmental samples, Analysis of microplastics by pyrolysis-GC/MS

## Abstract

This work describes the development of analytical workflows based on pyrolysis coupled with gas chromatography-mass spectrometry (Pyr-GC/MS) for the qualitative and quantitative analysis of 12 of the most common plastic polymers in environmental samples. The most suitable characteristic pyrolyzate compounds and respective indicator ions were selected for each polymer in order to obtain the most appropriate response for analytical purposes. Additionally, commercial pyrolyzates and polymers libraries were used to confirm the identity of the detected microplastics. The method was validated, showing a good linearity for all the plastic polymers (R^2^ > 0.97) and limits of detection between 0.1 (polyurethane) to 9.1 µg (polyethylene). The developed methodology was successfully applied for the analysis of plastic polymers in environmental microplastic samples collected in three Mediterranean beaches (NE Spain).

• Fast and reproducible Pyr-GC/MS method for the analysis of the 12 most common plastic polymers in a single GC/MS run
• Straightforward analytical workflows using pyrolyzates and polymers libraries enable a fast identification and quantification of microplastics in environmental samples

## Graphical abstract

**Figure:**
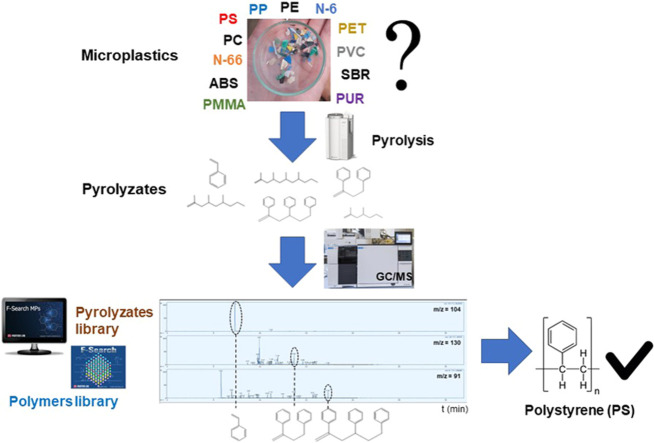

Specifications table

**Table:** 

Subject area:	Environmental Science
More specific subject area:	Microplastics analysis
Name of your method:	Analysis of microplastics by pyrolysis-GC/MS
Name and reference of original method:	N.A.
Resource availability:	Pyrolysis-GC/MS (Frontier Laboratories Ltd.; Agilent Technologies); F-Search software (Frontier Laboratories Ltd.); F-Search MPs software (Frontier Laboratories Ltd.); Pyrolyzate compounds library; Polymers library

## Method details

### Background

Due to their persistence, ubiquity, and toxic potential, microplastics, which are plastic fragments with a particle size smaller than 5 mm, have been recognized as an environmental threat and there is a growing concern about their presence and accumulation [1]. Microplastics can be either directly introduced into the environment or formed by natural degradation of larger plastics. There is a broad variety of synthetic polymers, which makes their analysis a very challenging task. To date several analytical techniques have been used for the analysis of microplastics in environmental samples. Although spectroscopic methods are among the most common approaches, allowing to get information on particle number, size, and polymer identification [2], pyrolysis coupled with gas chromatography-mass spectrometry (Pyr-GC/MS) has gained an increasing interest over the last years, because it allows both the identification and quantification of microplastics in complex environmental matrices [3]. Pyr-GC/MS is a destructive technique based on the thermal decomposition of polymers, which are then identified by the molecular profile of the generated degradation products [2,4].

In this work, analytical workflows based on Pyr-GC/MS were developed for the identification and quantification of 12 of the most common types of plastic polymers in a single GC/MS run. Data treatment was carried out using the software F-Search and F-Search MPs, which include pyrolyzates and polymers libraries, allowing a fast, reliable, and robust qualitative and quantitative analysis of microplastics. The proposed analytical workflows were successfully applied for the analysis of microplastics in samples collected in Mediterranean beaches of the province of Girona (NE Spain).

### Pyrolysis-GC/MS method

Microplastics analysis was performed by Pyr-GC/MS in a system equipped with a Multi-Shot EGA/PY-3030D micro-furnace pyrolyzer and an Auto-Shot sampler AS-1020E (Frontier Laboratories Ltd.) and a GC 8890 gas chromatograph with an MSD 5977B single quadrupole mass spectrometry detector (Agilent Technologies). Microplastics analysis was performed in single-shot mode. For that, the pyrolysis furnace temperature was set at 600 °C with a pyrolysis holding time of 0.2 min. The Pyr-GC transfer line was set at 300 °C. The pyrolysis was online coupled with the split/splitless (SSL) injector of the GC/MS system. The GC injector operated in split mode with a 50:1 ratio at a temperature of 300 °C. The pyrolyzates were analyzed under the following optimized conditions: the separation was carried out on an Ultra Alloy UA^+^- 5 capillary column (5% diphenyl-95% dimethylpolysiloxane) (30 m × 0.25 mm i.d. × 0.25 µm film thickness) from Frontier Laboratories Ltd. coupled with a post-column (deactivated fused silica 1 m × 0.1 mm) supplied by Agilent Technologies. Helium 6.0 of purity (Air Liquide) was used as carrier gas at a flow rate of 1.37 mL/min. The GC oven program was the following: 2 min at 40 °C, then increasing by 20 °C/min up to 320 °C and held for 16 min. The detector transfer line was set at 320 °C. The mass spectrometer detector was operated with an electron ionization source (EI) with a voltage of 70 eV, quadrupole temperature at 150 °C, and source temperature at 230 °C. The measurements were carried out in full scan mode with a *m/z* range of 29-400 and a scan rate of 2 scan/s.

### Preparation of pyrolysis cups

For the preparation of the pyrolysis cups, a microbalance with six decimals and a readability of 1 µg (XP6, Mettler Toledo) was used. A commercial mixture of the 12 most common plastic polymers, namely polystyrene (PS), polyethylene (PE), polypropylene (PP), polyvinylchloride (PVC), polyethylene terephthalate (PET), polycarbonate (PC), polyurethane (PUR), Nylon 6 (N-6), Nylon 66 (N-66), polymethyl methacrylate (PMMA), styrene-butadiene copolymer (SBR), and acrylonitrile butadiene styrene copolymer (SBR), prepared with CaCO_3_ as inert diluent (Frontier Laboratories Ltd.) was used. The exact mass of each plastic polymer present in the commercial mixture supplied by Frontier Laboratories Ltd. is indicated in Table S1 (Supplementary Material).

For the preparation of the calibration curves, different amounts (0.4, 1.0, 2.0, and 4.0 mg) of the mixture of 12 microplastics (MPs-CaCO_3_) were weighted to the pyrolysis cup and covered with quartz wool to prevent the loose of the mixture of microplastics standards during the introduction of the cup in the pyrolyzer. The quartz wool was introduced into the pyrolysis cup with the help of tweezers, guaranteeing that the sample was completely covered, and using an amount equivalent to 20% of the quantity of standard or sample weighted.

In the case of microplastics samples, aliquots of the collected samples (0.25 mg) were weighted to the pyrolysis cups and covered with quartz wool before analysis by Pyr-GC/MS. All the samples were prepared in triplicate.

### Sampling details

Microplastics visible to the naked eye (particles with 2-5 mm) were collected in three beaches of the province of Girona, Catalonia (NE Spain) in June 2022, namely Almadrava (Roses), Cala Montgó (l'Escala), and Riuet beach (Sant Pere Pescador). Twenty microplastic samples were randomly collected from the sand of each beach and were stored in glass Petri dishes. After arriving at the laboratory, a total of six microplastic samples were selected for further Pyr-GC/MS analysis. Each selected microplastic sample was cut in small pieces with a cutler and a 0.25 mg aliquot was weighted to a pyrolysis cup and analyzed by Pyr-GC/MS. The physical characterization of the microplastic samples selected for analysis is present in Table 1.

**Table 1:** Physical characterization of the analyzed microplastic samples.

Sample code	Sampling location (beach)	Size (mm)	Color	Shape
S1	Almadrava (Roses)	4	White	Spheric fragment
S2	Cala Montgó (l'Escala)	5	Green	Fragment
S3	Cala Montgó (l'Escala)	4	White	Fragment
S4	Cala Montgó (l'Escala)	4	Pink	Fragment
S5	Riuet (Sant Pere Pescador)	5	Yellow	Fragment
S6	Riuet (Sant Pere Pescador)	3	Blue	Fragment

### Polymer identification

Pyrolysis-GC/MS gives information on the thermally degraded volatile organic compounds derived from the pyrolysis of plastic polymers. Therefore, the definition of specific pyrolyzates and their respective characteristic indicator ions for each plastic polymer is needed for the identification and quantification of plastic polymers in environmental matrices. This is a crucial step, given that some pyrolyzate compounds can come from different sources, given that they can be shared by different plastic polymers and/or they can be present in natural substances (e.g., humic substances, lignins, etc.) [4]. Thus, a literature review was done to create a database of the characteristic pyrolyzates and respective indicator ions for the studied 12 plastic polymers. Then, a 2 mg standard mixture of plastic polymers (MPs-CaCO_3_) was analyzed under the Pyr-GC/MS conditions previously described. The obtained pyrograms were compared with the in-house database to identify the characteristic pyrolyzates, and the most abundant and/or polymer-specific compounds were chosen as the most suitable characteristic pyrolyzates, and respective indicator ions, for the identification and quantification of each polymer (Table 2).

**Table 2:** Characteristic pyrolyzate compounds of the 12 target plastic polymers, respective indicator ions and retention time under the defined Pyr-GC/MS conditions. *Pyrolyzate compounds used for quantification are indicated in italic.

Polymer	Pyrolyzate compounds	Indicator ion (m/z)	t_R_ (min)
PS	Styrene	104	7.37
	Styrene dimer: 3-butene-1,3-diyldibenzene	130	13.75
	Styrene trimer: 5-hexene-1,3,5-triyltribenzene*	*91*	17.43
PP	2,4-Dimethyl-1-heptene*	*70*	6.76
	2,4,6-Trimethyl-1-nonene	97	9.21
	2,4,6,8-Tetramethyl-1-undecene	69	10.90
PE	1,20-Heneicosadiene (C_21’’_)*	*82*	14.92
	1-Dodecene (C_12_)	83	10.03
	1-Hexadecene (C_16_)	83	11.5
PET	Benzene	78	4.63
	Benzoic acid	122	11.70
	Diphenyl	154	11.61
	Benzophenone*	*182*	13.24
PMMA	Methyl methacrylate*	*100*	5.17
	Methyl acrylate	55, 85	4.14
PVC	Toluene	91	5.90
	Naphthalene*	*128*	10.18
	Xylene	106	7.12
	Fluroene	166	13.04
N-6	Ɛ-caprolactam*	85, *113*	10.64
N-66	Cyclopentanone*	*84*	6.15
	1,8-Diazacyclotetradecane-2,7-dione	112	16.52
PC	Phenol	94	8.29
	p-Isopropenylphenol*	*134*	10.92
PUR	4,4‘-Methylenbis(N-methylaniline)*	*198*	15.92
ABS	2-Methylene-4-phenylbutanenitrile	91	8.02
	2-Phenethyl-4-phenylpent-4-enenitrile*	*170*	16.13
	2-Methylene-4-phenylheptanedinitrile	144	14.35
SBR	Styrene	104	7.37
	Butadiene dimers (C_12_H_18_)	91	9.84
	4-Phenylcyclohexene*	*104*	11.29

⁎ Pyrolyzate compounds used for quantification

### Data treatment

Data processing of the results of Pyr-GC/MS is a time-consuming step and most of the work must be done manually. Therefore, the development of analytical workflows that could include some degree of automation on the data processing for the identification and quantification of plastic polymers in complex environmental samples is valuable. In fact, automation of data processing is one of the challenges faced by Pyr-GC/MS analysis of microplastics [3]. Nowadays, there is software for data treatment that might help in the hard task of microplastic identification and quantification. In this work, analytical workflows for plastic polymer identification and quantification were established using F-Search and F-Search MPs, respectively (both from Frontier Laboratories Ltd.) taking advantage of the commercial libraries included in them.

### Analytical workflows for plastic polymer identification using F-Search software

**Fig. 1:**
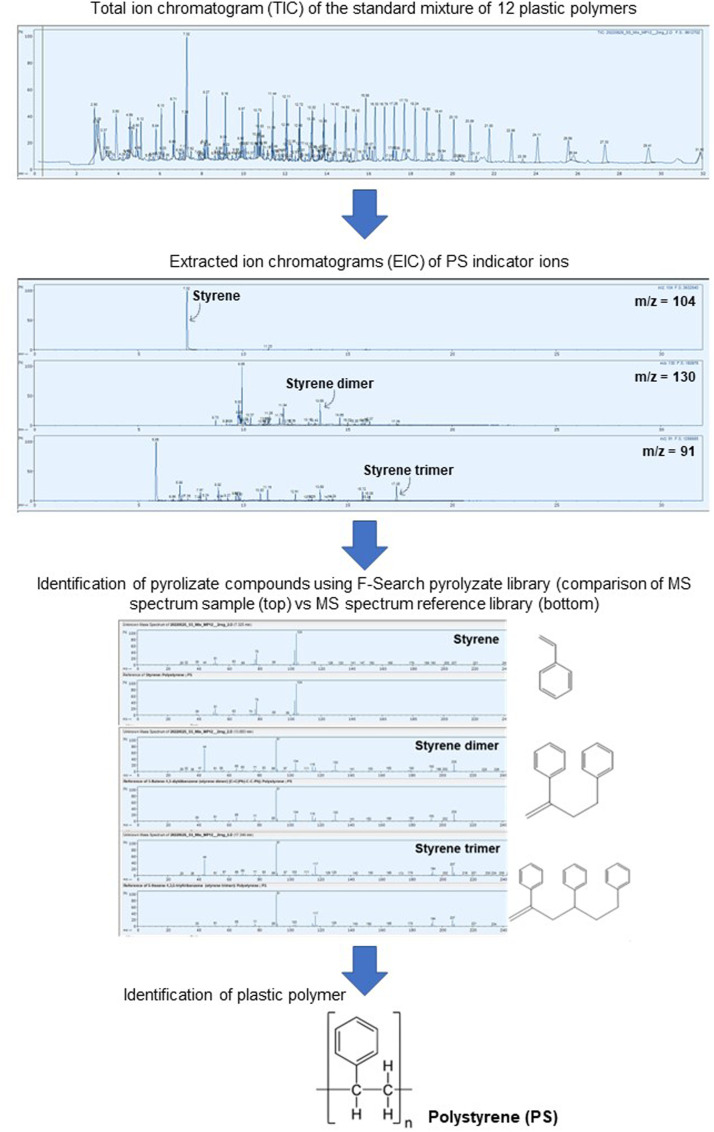
Example of the analytical workflow for the identification of polystyrene (PS) in the standard mixture of 12 plastic polymers using F-Search and its pyrolyzates library.

After Pyr-GC/MS analysis, the acquired pyrograms of the standard mixture and samples were processed for the identification of plastic polymers. Fig. 1 summarizes the analytical workflow followed for the identification of plastic polymers using F-Search (Frontier Laboratories Ltd.) and showing as an example the identification of PS in a 2 mg standard mixture of 12 plastic polymers (MPs-CaCO_3_). The analytical workflow consisted in the following steps:

(1) Upload the pyrogram of the standard mixture of 12 plastic polymers to F-Search.
(2) Detect the peaks present in the pyrogram.
(3) Obtain the extracted ion chromatograms (EIC) of the characteristic indicator ions of PS (styrene (m/z = 104), styrene dimer (m/z = 130), and styrene trimer (m/z = 91) – see Table 2).
(4) Select the peak of each characteristic pyrolyzate compound of PS based on the corresponding retention time (see Table 2).
(5) Identify the characteristic pyrolyzate compounds of PS (styrene, styrene dimer, and styrene trimer (Table 2)) by comparing the MS spectrum of each of the selected pyrolyzate compounds with the MS spectrum of reference compounds from the pyrolyzates library ("Pyrolyzate-MS18B library”) included in F-Search.
(6) A positive match for styrene, styrene dimer, and styrene trimer allows the identification of PS.

***Note #1:*** If necessary, the background noise can be subtracted in order to remove matrix interferences and get a clearer MS spectrum, facilitating the identification of the compounds of interest.

F-Search also allows the identification of plastic polymers in real samples, given that it has a polymers library incorporated that allows the identification of the most common plastic polymers. For that, the data processing consisted in the next steps:

(1) Upload the pyrogram of the sample to F-Search.
(2) Detect the peaks present in the pyrogram.
(3) Get the MS spectrum of the sample considering all the time range (mass spectrum that is the sum of the MS spectra of all detected pyrolyzate compounds).
(4) Identify the plastic polymer of the sample by searching the MS spectrum of the sample in the “PyGC-MS18B library” (a polymers library).
(5) Identification of the plastic polymer is done by comparison of the MS spectrum of the sample with the MS spectrum of reference from the polymers library. A matching score higher than 80% guarantees the identification of the plastic polymer [5].
(6) Complementarily, the identity of the plastic polymer can be confirmed using its characteristic pyrolyzate compounds and respective retention times, as previously described.

**Fig. 2:**
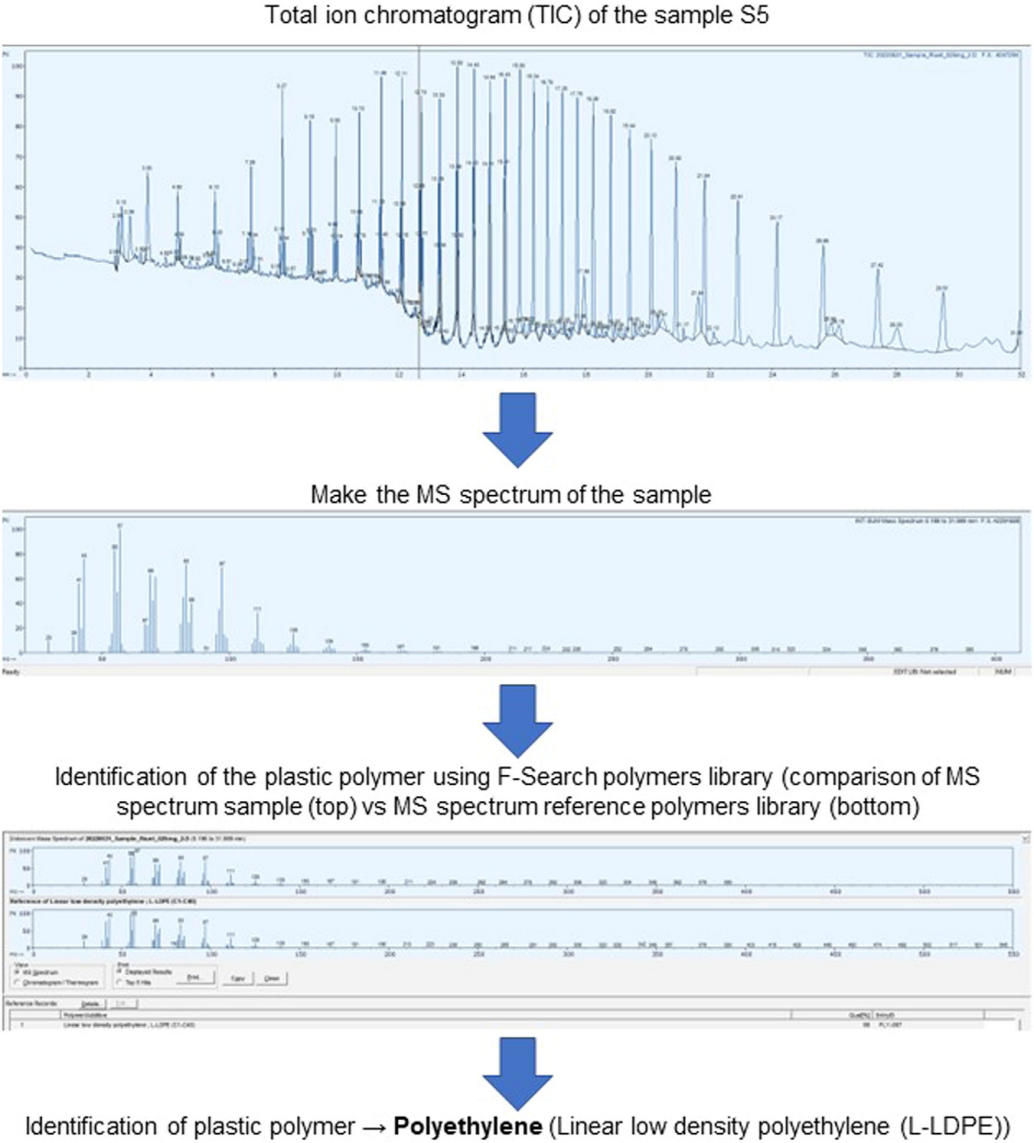
Example of the analytical workflow for the identification of the plastic polymer in the microplastic sample S5, collected on the Riuet beach (see Table 1), using F-Search and its polymers library.

In this study, a total of six samples of microplastics visible to the naked eye, which were collected in three Mediterranean beaches (NE Spain), were selected for Pyr-GC/MS analysis. Fig. 2 shows an example of the application of the analytical workflow for the identification of the plastic polymer in sample S5 (Table 1), collected in the Riuet beach, using F-Search. The identification of the plastic polymers for the remaining samples can be found in Supplementary Material (Figs. S1–S5).

### Analytical workflow for plastic polymer quantification using F-Search MPs software

For the quantification of plastic polymers in the environmental samples, an analytical workflow using F-Search MPs (Frontier Laboratories Ltd.) was established. The first step is to prepare a calibration curve for each plastic polymer. For that, four concentrations of the standard mixture of 12 plastic polymers (0.4, 1.0, 2.0, and 4.0 mg) were prepared in triplicate and analyzed by Pyr-GC/MS. Then, the obtained pyrograms were uploaded to F-Search MPs to prepare the calibration curves that would be further used for the quantification of plastic polymers in real samples.

**Fig. 3:**
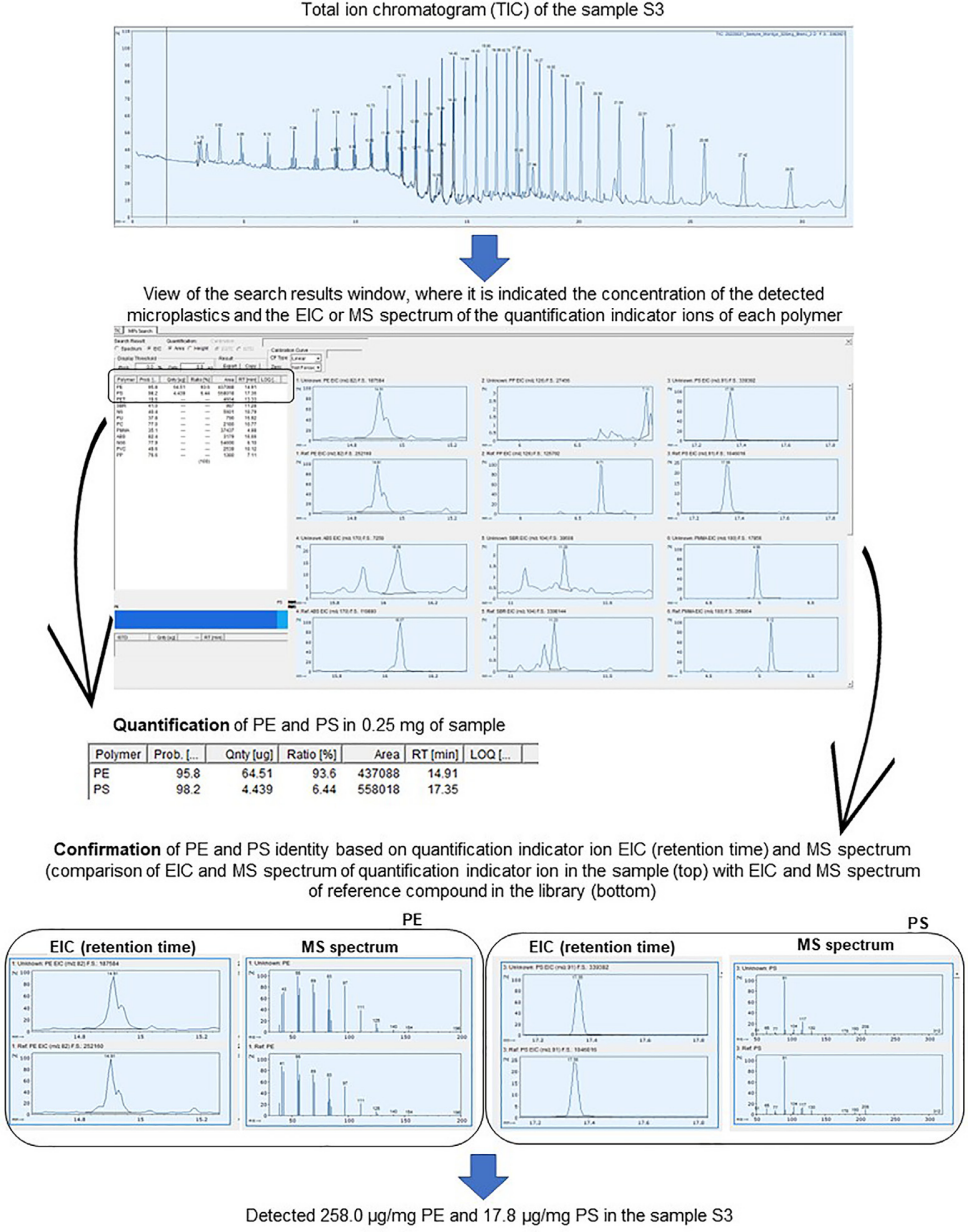
Example of the analytical workflow for the quantification of plastic polymers in a microplastic sample S3, collected on the Cala Montgó beach, using F-Search MPs.

Fig. 3 illustrates the analytical workflow used for the quantification of plastic polymers in the collected samples and that consisted in the following steps:

1. Upload the pyrogram of the sample to F-Search MPs.
2. Load the calibration curve previously prepared with the standard mixture of 12 microplastics to be used in the quantification of plastic polymers.
3. Detect the peaks present in the real sample pyrogram.
4. Search microplastics with F-Search MPs.
5. A summary table is created by the software with the results: concentrations (in micrograms) of the detected microplastics as well as the EIC and MS spectrum of the characteristic pyrolyzate compounds selected for quantification of each plastic polymer.
6. Validate the obtained results by verifying the retention time and MS spectrum of the characteristic pyrolyzate compound used for quantification. F-Search MPs allows the comparison of the EIC (retention time) and MS spectrum of the quantification indicator ion in the sample with those from the reference compound present in the library. A matching score higher than 80% and an amount of microplastic higher than the LOQ guarantee the positive identification of a plastic polymer in the sample [5].

***Note #2:*** The characteristic pyrolyzate compound of each plastic polymer used for quantification by F-Search MPs are indicated in Table 2. These compounds are already defined in the software, and they cannot be changed.

***Note #3:*** In the case that the identification results of the plastic polymer obtained with F-Search MPs are not clear, its identification can be confirmed using all the characteristic pyrolyzate compounds selected for a given polymer (Table 2), and respective retention times, and following the analytical workflow previously described for the identification of plastic polymers using F-Search (Fig. 1).

### Validation parameters

The validation of the Pyr-GC/MS method was done in terms of linearity, limit of detection (LOD), limit of quantification (LOQ), repeatability, and reproducibility. The limits of detection (LOD) and quantification (LOQ) were estimated for each plastic polymer using the corresponding calibration curves and applying Eqs. (1) and (2), respectively:

(1) $$\text{LOD}=3.3\times \frac{\sigma }{S}$$

(2) $$\text{LOQ}=10\times \frac{\sigma }{S}$$

where σ is the standard deviation of the response and *S* is the slope of the calibration curve.

The repeatability (intra-day precision) and reproducibility (inter-day precision) were evaluated using two different amounts of standard mixture of microplastics (MPs-CaCO_3_) (0.5 and 2.0 mg). For the repeatability, seven replicates of each amount of microplastics were analyzed in the same day, while for the reproducibility, three replicates of each amount of microplastics were analyzed in three consecutive days. The validation results obtained are summarized in Table 3.

**Table 3:** Validation parameters of the 12 target plastic polymers.

Polymer	Characteristic pyrolyzate	Indicator ion (m/z)	R^2^	LOD (µg)	LOQ (µg)	Intra-day precision (%RSD)		Inter-day precision (%RSD)	
						0.5 mg	2.0 mg	0.5 mg	2.0 mg
PS	Styrene trimer	91	0.9679	0.6	1.7	6.29	4.97	19.3	9.82
PP	2,4-Dimethyl-1-heptene	70	0.9896	2.2	6.6	3.21	2.45	8.56	6.02
PE	1,20-Heneicosadiene (C21’’)	82	0.9892	9.1	27.8	14.0	4.18	22.7	5.45
PET	Benzophenone	182	0.9968	0.9	2.9	9.49	4.03	18.7	10.4
PMMA	Methyl methacrylate	100	0.9788	0.5	1.4	4.00	1.91	10.5	6.72
PVC	Naphthalene	128	0.9952	1.7	5.3	4.31	2.10	7.67	6.91
N-6	Ɛ-caprolactam	113	0.9939	0.2	0.7	4.97	3.07	7.09	6.71
N-66	Cyclopentanone	84	0.9787	1.8	5.6	5.31	1.57	7.20	4.48
PC	p-Isopropenylphenol	134	0.9945	0.2	0.7	2.95	2.27	5.09	5.41
PUR	4,4‘-Methylenbis(N-methylaniline)	198	0.9882	0.1	0.4	12.2	5.33	38.1	12.9
ABS	2-Phenethyl-4-phenylpent-4-enenitrile	170	0.9942	0.5	1.7	9.61	3.06	18.3	8.96
SBR	4-Phenylcyclohexene	104	0.9771	0.2	0.7	3.38	2.82	6.48	6.49

Overall, the performance of the Pyr-GC/MS method was very satisfactory. A good linearity range was obtained for all the plastic polymers (coefficient R^2^ > 0.97). LODs and LOQs varied from 0.1 to 9.1 µg and from 0.4 to 27.8 µg, respectively. The method precision, expressed in %RSD, was, in general, below 20%.

### Analysis of environmental microplastic samples

The six selected microplastics samples collected in the beaches of the province of Girona (NE Spain) were analyzed by Pyr-GC/MS. Then, the described analytical workflows using F-Search and F-Search MPs were applied for the identification and quantification of the plastic polymers present in the samples. PS, PE, and PP were the only plastic polymers detected in the samples. The mean concentration of the detected plastic polymers in the analyzed samples is indicated in Table 4. The values are normalized by the amount of weighted sample.

**Table 4:** Mean concentration of the plastic polymers, expressed in µg/mg, in the microplastic samples collected in the beaches of the province of Girona (NE Spain).

Sample code	Sampling location (beach)	PS (µg/mg)	PE (µg/mg)	PP (µg/mg)
S1	Almadrava	219.6 ± 15.2	—	—
S2	Cala Montgó	5.02 ± 3.10	369.6 ± 12.2	—
S3	Cala Montgó	30.0 ± 32.0	249.8 ± 9.7	—
S4	Cala Montgó	—	—	70.1 ± 15.6
S5	Riuet	—	331.5 ± 33.2	—
S6	Riuet	41.4 ± 40.7	—	89.2 ± 15.6

Overall, the described Pyr-GC/MS methodology showed a good performance for the simultaneous identification and quantification of 12 of the most common plastic polymers in microplastics samples. Furthermore, the use of the commercial software F-Search and F-Search MPs, which include pyrolyzates and polymers libraries, allowed the establishment of simple and useful analytical workflows that enabled a fast identification and quantification of plastic polymers in environmental samples.

## Acknowledgments

This work was funded by the Spanish State Research Agency of the Spanish Ministry of Science and Innovation through the project ReUseMP3 (PID2020-115456RB-I00 /MCIN/AEI/10.13039/501100011033). G. Buttiglieri acknowledges Spanish State Research Agency of the Spanish Ministry of Science, Innovation and Universities for the Grant to the Creation of a permanent position Ramon y Cajal 2014 (RYC-2014-16754). The pyrolizer coupled to a Gas Chromatograph with a single quadrupole Mass Spectrometry (Py-GC/MS), Pyr EGA/PY-3030D, Frontier, GC 8890 MS5977B Inert Plus, Agilent facility received support from the CERCA Institute through the CERCAGINYS program, funded by the Spanish Ministry of Science and Innovation. The authors acknowledge the support of the Economy and Knowledge Department of the Catalan Government through a Consolidated Research Group (ICRA-ENV-2021 SGR 01282 and ICRA-TECH-2021 SGR 01283).

## CRediT authorship contribution statement

**Lúcia H.M.L.M. Santos:** Conceptualization, Methodology, Software, Visualization, Writing – original draft. **Sara Insa:** Conceptualization, Methodology, Software, Writing – review & editing. **Marta Arxé:** Investigation, Software, Validation. **Gianluigi Buttiglieri:** Funding acquisition, Writing – review & editing. **Sara Rodríguez-Mozaz:** Funding acquisition, Writing – review & editing. **Damià Barceló:** Funding acquisition, Writing – review & editing.

## Declaration of Competing Interest

The authors declare that they have no known competing financial interests or personal relationships that could have appeared to influence the work reported in this paper.

## Data Availability

Data will be made available on request.

## Appendix Supplementary materials

Image, application 1
