# Supplementary material for: Analysis of microplastics in the environment: Identification and quantification of trace levels of common types of plastic polymers using pyrolysis-GC/MS
Source: MethodsX. 2023 Mar 21;10:102143. doi: 10.1016/j.mex.2023.102143 (PMC10050779; doi:10.1016/j.mex.2023.102143)
Supplement: Supplementary file 1 [file mmc1.docx]

**Supplementary Material for**

**Analysis of microplastics in the environment: Identification and quantification of trace levels of common types of plastic polymers using pyrolysis-GC/MS**

Lúcia H.M.L.M. Santos^a,b,*^, Sara Insa^a,b^, Marta Arxé^a,b^, Gianluigi Buttiglieri^a,b^, Sara Rodríguez-Mozaz^a,b,*^, Damià Barceló^a,b,c^

^a^Catalan Institute for Water Research (ICRA-CERCA), C/ Emili Grahit 101, 17003 Girona, Spain

^b^University of Girona, Girona, Spain

^c^IDAEA-CSIC, Department of Environmental Chemistry, C/ Jordi Girona 18-26, 08034 Barcelona, Spain

Corresponding author: Lúcia H.M.L.M. Santos (email address: [lhsantos@icra.cat](mailto:lhsantos@icra.cat)); Sara Rodriguez-Mozaz (email address: srodriguez@icra.cat)

**Table S1 –** Amount of each plastic polymer present in the commercial mixture of microplastics. In the commercial mixture, the 12 microplastics were diluted in CaCO_3_. The quantities indicated below refer to the amount of each plastic polymer in 4 mg of commercial MPs-CaCO_3_ powder (quantities provided by the supplier).

| **Plastic polymer** | **Quantity (µg)** |
| --- | --- |
| Polystyrene (PS) | 5.2 |
| Polyethylene (PE) | 145.8 |
| Polypropylene (PP) | 35.7 |
| Polyvinylchloride (PVC) | 42.4 |
| Polyethylene terephthalate (PET) | 28.6 |
| Polycarbonate (PC) | 5.1 |
| Polyurethane (PU) | 2.1 |
| Nylon 6 (N-6) | 4.9 |
| Nylon 6,6 (N-66) | 20.7 |
| Polymethyl methacrylate (PMMA) | 5.3 |
| Styrene-butadiene copolymer (SBR) | 15.0 |
| Acrylonitrile butadiene styrene copolymer (ABS) | 12.1 |


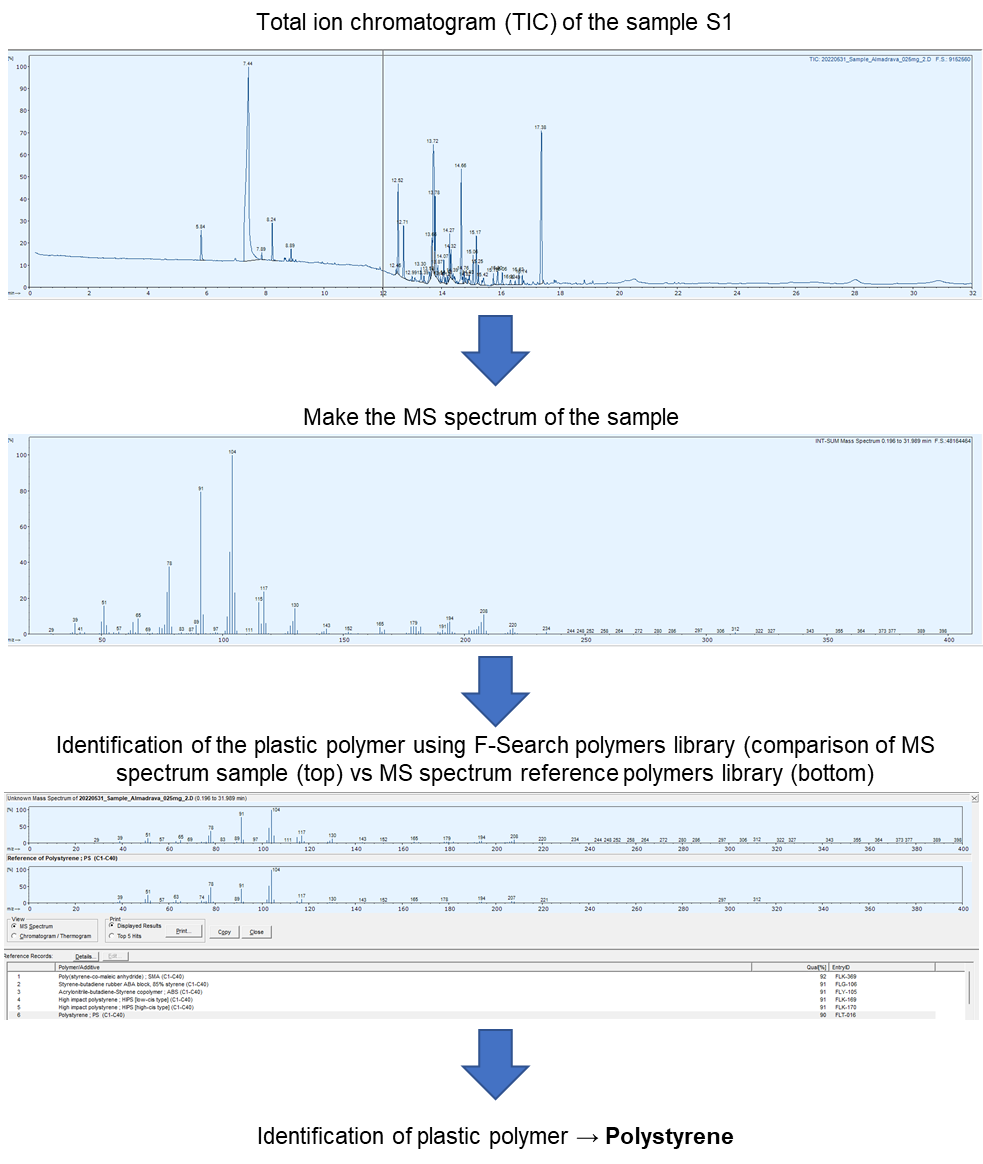


**Figure S1 -** Analytical workflow for the identification of the plastic polymer in the microplastic sample S1, collected in Almadrava beach, using F-Search and its polymers library.


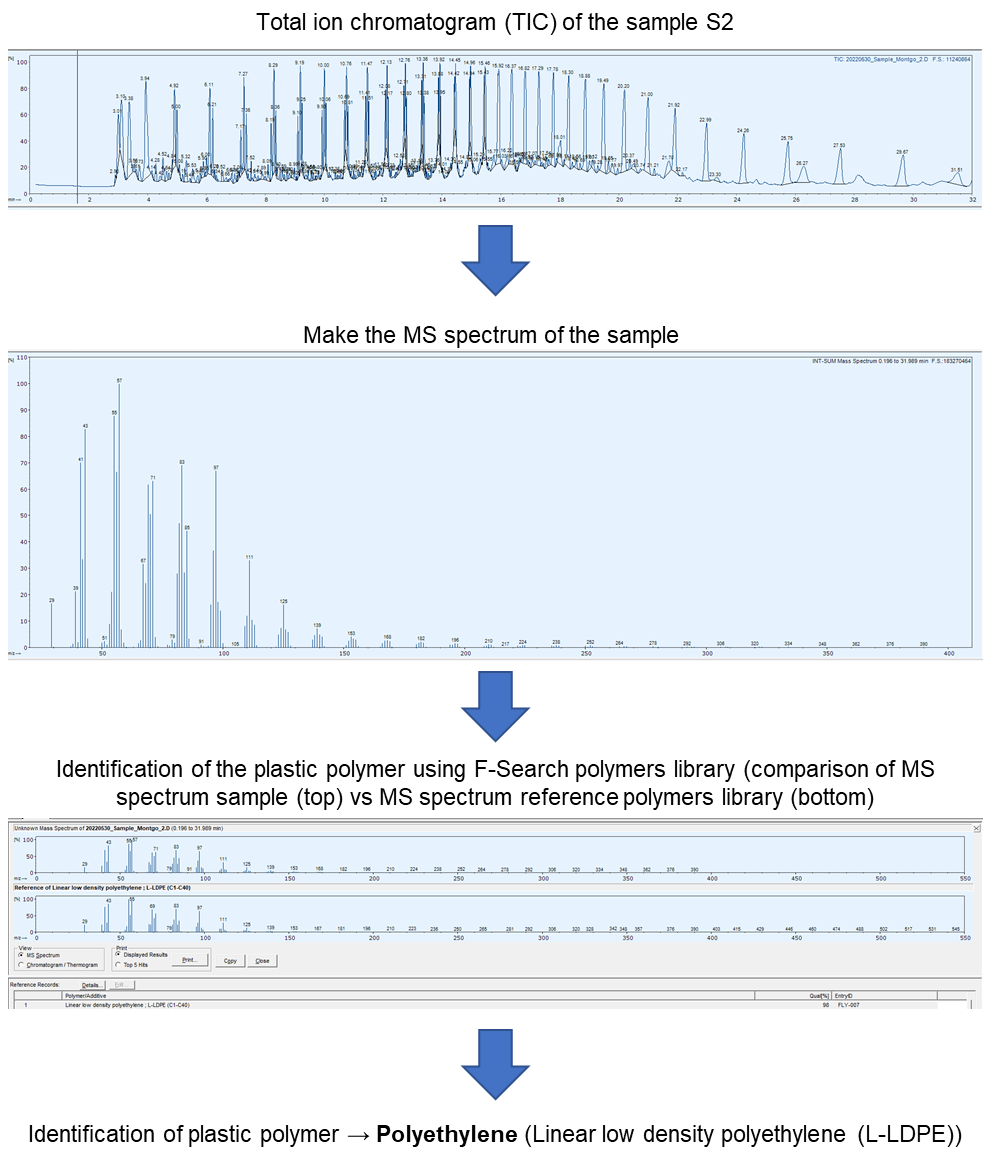


**Figure S2 -** Analytical workflow for the identification of the plastic polymer in the microplastic sample S2, collected in Cala Montgó beach, using F-Search and its polymers library.


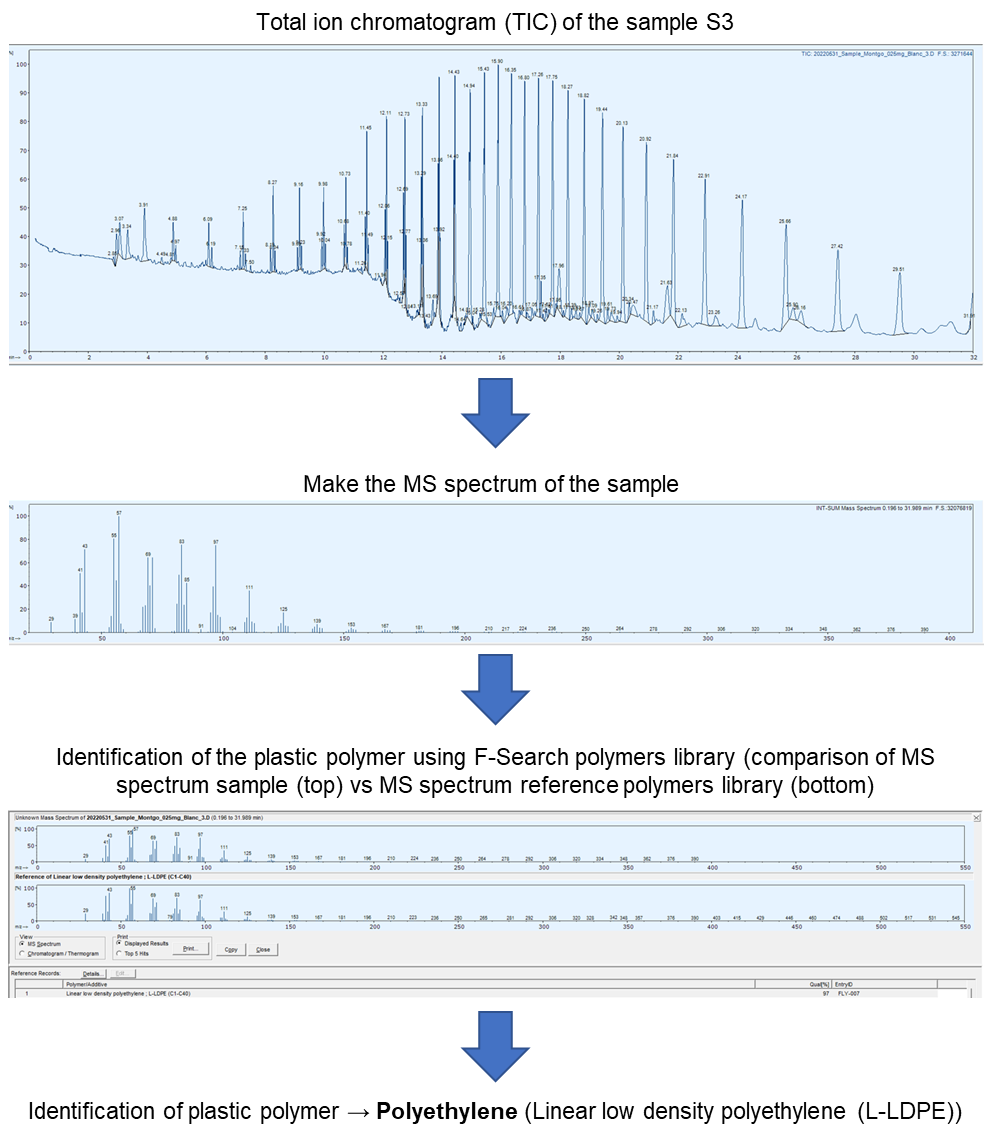


**Figure S3 -** Analytical workflow for the identification of the plastic polymer in the microplastic sample S3, collected in Cala Montgó beach, using F-Search and its polymers library.


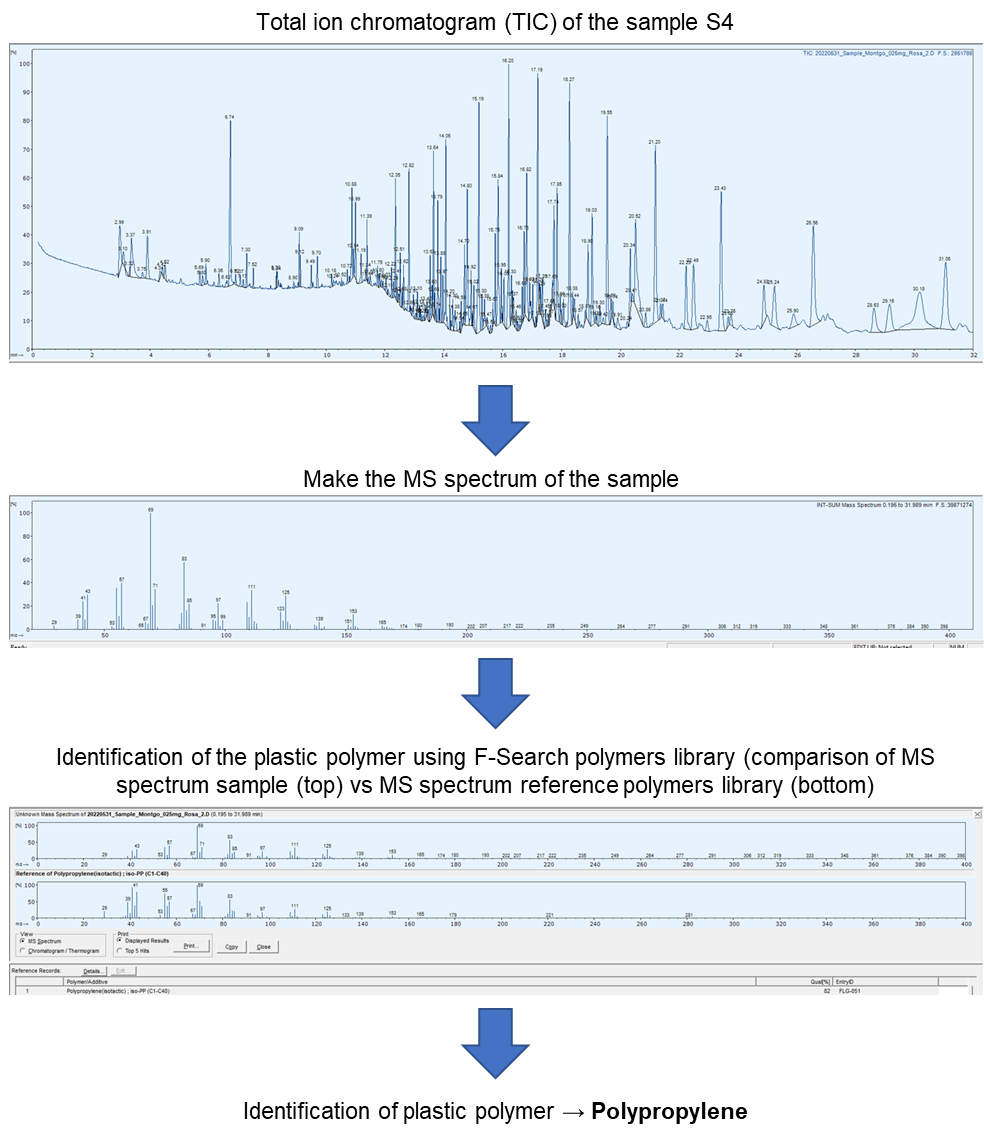


**Figure S4 -** Analytical workflow for the identification of the plastic polymer in the microplastic sample S4, collected in Cala Montgó beach, using F-Search and its polymers library.


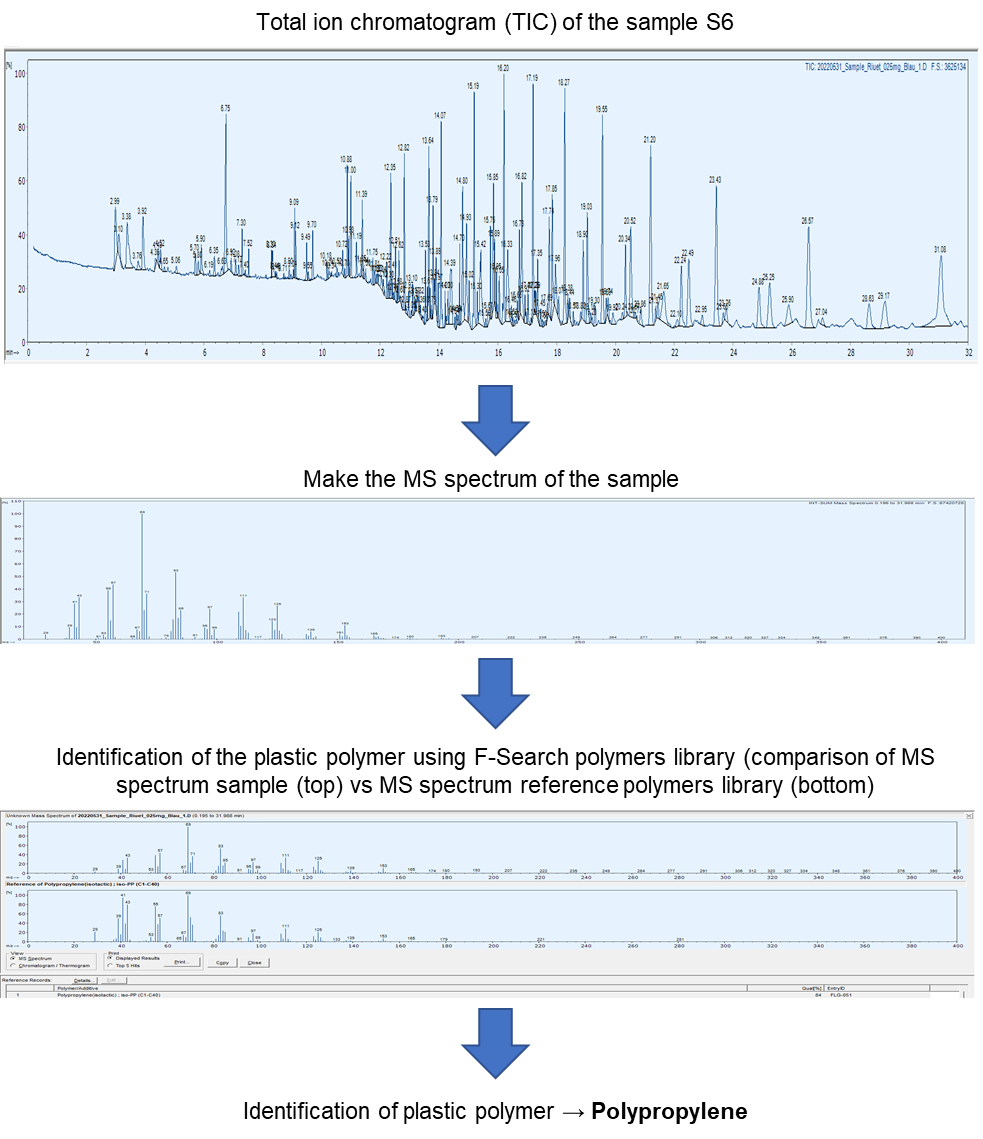


**Figure S5 -** Analytical workflow for the identification of the plastic polymer in the microplastic sample S6, collected in Riuet beach, using F-Search and its polymers library.
